# WHIRLY2 plays a key role in mitochondria morphology, dynamics, and functionality in *Arabidopsis thaliana*

**DOI:** 10.1002/pld3.229

**Published:** 2020-05-30

**Authors:** Serena Golin, Yuri L. Negroni, Bationa Bennewitz, Ralf B. Klösgen, Maria Mulisch, Nicoletta La Rocca, Francesca Cantele, Gianpiero Vigani, Fiorella Lo Schiavo, Karin Krupinska, Michela Zottini

**Affiliations:** ^1^ Department of Biology University of Padova Padova Italy; ^2^ Institute of Biology‐Plant Physiology Martin Luther University Halle‐Wittenberg Halle (Saale) Germany; ^3^ Institute of Botany Christian‐Albrechts University of Kiel Kiel Germany; ^4^ Department of Chemistry University of Milano Milano Italy; ^5^ Department of Life Science and Systems Biology University of Turin Turin Italy

**Keywords:** *Arabidopsis thaliana*, mitochondria, nucleoid, seed germination

## Abstract

WHIRLY2 is a single‐stranded DNA binding protein associated with mitochondrial nucleoids. In the *why 2‐1* mutant of *Arabidopsis thaliana*, a major proportion of leaf mitochondria has an aberrant structure characterized by disorganized nucleoids, reduced abundance of cristae, and a low matrix density despite the fact that the macroscopic phenotype during vegetative growth is not different from wild type. These features coincide with an impairment of the functionality and dynamics of mitochondria that have been characterized in detail in wild‐type and *why 2‐1* mutant cell cultures. In contrast to the development of the vegetative parts, seed germination is compromised in the *why 2‐1* mutant. In line with that, the expression level of *why 2* in seeds of wild‐type plants is higher than that of *why 3*, whereas in adult plant no difference is found. Intriguingly, in early stages of shoots development of the *why 2‐1* mutant, although not in seeds, the expression level of *why 3* is enhanced. These results suggest that WHIRLY3 is a potential candidate to compensate for the lack of WHIRLY2 in the *why 2‐1* mutant. Such compensation is possible only if the two proteins are localized in the same organelle. Indeed, *in organello* protein transport experiments using intact mitochondria and chloroplasts revealed that WHIRLY3 can be dually targeted into both, chloroplasts and mitochondria. Together, these data indicate that the alterations of mitochondria nucleoids are tightly linked to alterations of mitochondria morphology and functionality. This is even more evident in those phases of plant life when mitochondrial activity is particularly high, such as seed germination. Moreover, our results indicate that the differential expression of *why 2* and *why 3* predetermines the functional replacement of WHIRLY2 by WHIRLY3, which is restricted though to the vegetative parts of the plant.

## 1 INTRODUCTION

Mitochondria occupy a central place in the metabolic network of eukaryotic cells, with essential metabolic processes occurring within the organelle itself and several other pathways either emanating from or converging on mitochondria. Mitochondria can form dynamic, interconnected networks, regulated by a dynamic equilibrium between fusion and fission events that in turn determine their number, size, shape and functionality. A high motility of mitochondria is required to encounter different energy requirement of the cells in different developmental stages or environmental conditions (Zottini, Barizza, Bastianelli, Carimi, & Lo Schiavo, 2006). The regulation of mitochondrial shape dynamics plays a critical role in energy homeostasis as it responds rapidly and directly to acute metabolic perturbations contributing to energy demand and homeostasis (Yu & Pekkurnaz, 2018). Alterations of the dynamics and shape of mitochondria are also linked to genome instability (Xu et al., 2011). The balance between fission and fusion of mitochondria ensures the integrity of the organelle genome and the equal distribution of DNA among the mitochondria (Arimura, 2018). It has been observed that in the regions of the plant where a high cell division occurs, e.g., in germinating seeds and in shoot apical meristems, mitochondria have an elongated shape due to the dominance of the fusion over the fission process, coincident with an active mtDNA synthesis. Mitochondrial fusion provides indeed an opportunity for recombination of mtDNA fragments occurring during the replication of mtDNA (Arimura, 2018).

WHIRLY2 belongs to a small family of ssDNA binding proteins characteristic for higher plants (Desveaux, Allard, Brisson, & Sygusch, 2002). All WHIRLY proteins have in common the highly conserved WHIRLY domain including the KGKAAL motif implicated in binding to ssDNA (Desveaux, Maréchal, & Brisson, 2005). The crystal structure of the WHIRLY domain was determined by X‐ray diffraction analysis (Cappadocia et al., 2010). Such analyses revealed that tetrameric WHIRLIES bind to ssDNA in a sequence unspecific manner. By atomic force microscopy, it has been shown that hexamers of WHIRLY2 tetramers assemble into 24‐meric higher‐order structures upon binding long DNA molecules, whereby the interactions between the tetramers depend on K67 within the KGKAAL motif (Cappadocia, Parent, Sygusch, & Brisson, 2013). The structure of WHYRLY domain is highly conserved among and different WHIRLY proteins and plants, as revealed by a 3D structure analysis (Akbudak & Filiz, 2019). WHIRLY2, together with other organellar ssDNA binding proteins, plays a key role in the maintenance of integrity of mitochondrial DNA that is an absolute requirement for cell growth and proliferation. Failure in maintaining the stability of the mitochondrial genome would result in the accumulation of mutations and genomic rearrangements that can become deleterious (Gualberto & Kühn, 2014).

While most plants possess two WHIRLY proteins, *Arabidopsis thaliana* and other members of the *Brassicaceae* family have three WHIRLY proteins, which show differential organelle targeting (Krause et al., 2005). WHIRLY1 was imported into chloroplasts, both in *in organello* experiments and after transient expression in protoplasts. WHIRLY2 was instead imported into mitochondria in the transient expression analysis but showed dual targeting into both isolated organelles, chloroplasts and mitochondria. WHIRLY3, on the other hand, was solely analyzed with transient expression assays and showed targeting of the GFP reporter to chloroplasts (Krause et al., 2005).

Arabidopsis plants, lacking either plastid or mitochondrial WHIRLY proteins, accumulate higher levels of microhomology‐mediated DNA rearrangements (MHMRs) than wild‐type (Cappadocia et al., 2010;Maréchal et al., 2008) indicating that WHIRLY proteins act as components of the organellar DNA repair machinery. It has been proposed that WHIRLY2 prevents the accumulation of abnormal mtDNA molecules by limiting the microhomology‐mediated end‐joining during double‐stranded breaks in mtDNA repair process (García‐Medel et al., 2019).

Under standard growth conditions, adult plants of the Arabidopsis *why 2‐1* mutant do not show any obvious difference in the phenotype compared to the wild type (Maréchal et al., 2008). However, overexpression of *AtWHIRLY2* in Arabidopsis causes a reduction in mitochondrial transcripts and mitochondrial DNA, translating into lower activities of the respiratory chain complexes as well as earlier senescence (Maréchal et al., 2008). Since WHIRLIES are evidently associated with organellar DNA, tissues having low levels of mtDNA, such as mature pollen of Arabidopsis, lack expression of WHIRLY2 (Cai, Guo, Shen, Wang, & Zhang, 2015). On the other hand, overexpression of *WHIRLY2*, under control of a promoter that is specifically active in pollen vegetative cells, leads to slower growth of pollen tubes paralleled by an increase in mtDNA content of pollen and accumulation of reactive oxygen species (Cai et al., 2015).

Apart from its role in maintaining the integrity of mtDNA and organellar gene expression, information about the role of WHIRLY2 in the modulation of mitochondrial metabolism and morphology is still lacking.

Therefore, in this study, transgenic Arabidopsis plants as well as cultured cells defective in *AtWHIRLY2* expression (*why 2‐1* mutant lines) were used to investigate at the cellular level, the impact of WHYRLY2 on the mitochondrial morphology, dynamics and function. Furthermore, we investigate whether the presence of aberrant mitochondria in the *why 2‐1* mutant could have consequences for development of the plant. And, finally, we provide indications based on gene expression and immunological analyses together with organelle import assays that the lack of WHYRLY2 in mitochondria can be compensated by WHIRLY3 in a tissue‐specific manner.

## 2 MATERIAL AND METHODS

### 2.1 Plant materials and growth conditions

All experiments were performed using the Arabidopsis (*A. thaliana*) ecotype Columbia (Col‐0). The T‐DNA insertion mutant line *why 2‐1* (SALK_118900), obtained from the Nottingham Arabidopsis Stock Centre (http://arabidopsis.org), corresponds to a line previously described as a true knockout mutant (Janicka et al., 2012). T‐DNA insertion sites were determined by PCR (*primer WHY2 For 5’‐GCATCCTCAAAACCAATGAC‐3ʹ*, *primer WHY2 Rev 5ʹ‐CATGATGTGTGGAAGAGCAA‐3ʹ*, and *primer T‐DNA Rev 5ʹ‐ATTTTGCCGATTTCGGAAC‐3ʹ*) and subsequent sequencing.

The seeds were surface sterilized in 70% (v/v) EtOH and 0.05% Triton X‐100, followed by pure EtOH. The seeds were sown onto square Petri dishes containing one‐half MS medium (Murashige & Skoog, 1962) supplemented with 0.5 g/L MES‐KOH, pH 5.8, 0.8% (w/v) Plant Agar (Duchefa), and 1% (w/v) Suc, stratified for 2 days at 4°C in the dark, and placed vertically in a growth chamber at 22°C with 16‐hr day length and PAR of 150 μmol m^−2^ s^−1^. The experiments were conducted at different Arabidopsis growth stages, as defined by Boyes et al. (2001).

Some experiments were conducted with non‐embryogenic cell suspension cultures of wild type (WT) and *why 2‐1* lines. Suspension cell cultures were grown at 25°C under a long daylight period on a gyratory shaker in liquid MSR2 medium (MS medium supplemented with 2 mg/L glycine; 0.5 mg/L nicotinic acid; 0.1 mg/L thiamine hydrochloride; 0.5 mg/L pyridoxal hydrochloride; 100 mg/L myo‐Inositol; 0.5 g/L malt extract; 3% (w/v) Suc; 1 mg/L 6‐BAP; and 2 mg/L 2,4‐D; pH 5.8). For subculturing, 2 ml of packed cells were transferred into 50 ml fresh medium every 7 days. To determine the growth capabilities of the two suspension cultures, cells were filtered and their dry weight was determined. For the dry weight, the cells were placed in a stove for 24 hr at 40°C. Experiments were performed at 5 days after subcultivation, when cells are in the exponential growth phase.

For the *in organello* protein transport experiments, pea seedlings (*Pisum sativum* var. Feltham First) were grown on soil for 7 days at a 16 hr photoperiod under constant temperature (18–22°C).

### 2.2 Oxygen consumption measurements

Oxygen consumption was measured using a Clark‐type oxygen electrode (Hansatech Instrument, United Kingdom). Respiration of Arabidopsis cell suspension cultures was measured in the dark at 25°C. One ml of a 5‐day‐old suspension cell culture was placed in the chamber containing 1 ml of MSR2 medium.

### 2.3 RNA isolation and qRT‐PCR

Plants and cells of the WT and *why 2‐1* mutant lines were harvested for subsequent analyses. Total RNA was extracted from samples using RNeasy^®^ Plant Mini Kit (Qiagen) according to the manufacturer's instructions. RNA concentration was measured using a Nanodrop ND‐1000 spectrophotometer (Nanodrop Technologies). First‐strand cDNA synthesis was performed using 2 μg of RNA, oligo(dT) primers, and SuperScript‐II Reverse Transcriptase (Invitrogen) according to the manufacturer's instructions. The qRT‐PCR reactions were performed with 100 ng of cDNA using the SYBR Green technology of Go Taq^®^ qPCR Master Mix (Promega) in a 7500 Real‐time PCR System (Life Technologies). The primers sequences for qRT‐PCR are reported in Table S1. The expression levels of each gene were normalized to the expression level of the housekeeping gene *ACTIN‐2* (*ACT2*; At3g18780) and analyzed using the ΔΔCT method (Livak & Schmittgen, 2001).

### 2.4 Confocal laser scanning microscopy

Confocal Laser Scanning Microscopy (CLSM) analyses were performed using a Zeiss LSM700 (Carl Zeiss Microscopy). Cells and seedlings were incubated for 20 min in 0.25 μM of tetramethylrhodamine (TMRM) solution. Samples were washed twice (10 mM MES, 10 mM CaCl_2_, and 5 mM KCl pH 5.8) and then observed. For TMRM detection samples were excited at 535 and fluorescence was measured at 600 nm. For GFP detection, excitation was at 488 nm and emission between 515/530 nm. For the chlorophyll detection, excitation was at 488 nm and detection over 570 nm. For PI detection, excitation was set at 548 nm and emission 573 nm. Acquired images were analyzed using the Fiji—ImageJ bundle software (http://fiji.sc/Fiji). The experiments were performed at least in triplicate, and each sample set comprised 10 samples.

### 2.5 Transmission electron microscopy

For ultrastructural analysis, small samples of the first leaves from WT and *why 2‐1* mutant were fixed in 2.5% (w/v) glutaraldehyde and 1% (v/v) formaldehyde (prepared from paraformaldehyde) in 0.1 M sodium cacodylate (pH 7.4) at 4°C overnight, and postfixed for 4 hr in buffered 1% osmium tetroxide on ice. Washing was done with 0.1 M sodium cacodylate (pH 7.4). The specimens were dehydrated in a graded series of ethanol and embedded in LR White resin (London Resin Company). Polymerization was in gelatine capsules at 60°C for 48 hr. Ultrathin sections of the specimens were cut with a diamond knife at a Leica Ultracut UCT ultramicrotome and placed on formvar‐coated copper grids. Sections were stained with uranyl acetate and with lead citrate (Reynolds, 1963), and subsequently observed in a Philips CM10 transmission electron microscope. Two replicates were analyzed for both WT and *why 2‐1* and 30–50 images were analyzed for each replicate.

### 2.6 Electron tomography

The tomography acquisitions were performed using Zeiss LIBRA 200FE‐HR TEM, operating at 200 kV and equipped with an in‐column omega filter for energy selective imaging and diffraction. The tomographic series were collected with a Fischione 2040 Dual‐Axis Tomography Holder, following the dual‐axis strategy. The 3D reconstruction is obtained by using weighted back‐projection algorithm and simultaneous alignment method followed by local refinement, as previously described (Vigani et al., 2015). The 3D reconstruction is obtained by use of weighted back‐projection algorithm and simultaneous alignment method according to Cantele, Paccagnini, Pigino, Lupetti, and Lanzavecchia (2010). Segmentation was carried out by using the program JUST to obtain the final tomogram of mitochondrial selected (Salvi et al., 2008). The tracings from all sections are modeled as 3D surfaces and displayed as a 3D model by the program Avizo (FEI, SAS).

### 2.7 In organello protein transport assay

Radiolabeled precursor proteins of AtWHIRLY3 and the organelle‐specific control proteins FNR (chloroplast Ferredoxin‐NADP‐Oxidoreductase) and mitochondrial Rieske Fe/S protein were obtained by in vitro translation in rabbit reticulocyte lysates in the presence of [35S]‐methionine. Incubation with intact mitochondria or chloroplasts isolated from pea leaves followed the protocol of Rödiger, Baudisch, and Bernd Klösgen (2010). Competition experiments were performed as described (Bennewitz, Sharma, Tannert, & Klösgen, 2020).

Gel electrophoresis of proteins under denaturing conditions was carried out according to Laemmli (1970). The gels were exposed to phosphorimaging screens and analyzed with a Fujifilm FLA‐3000 (Fujifilm) using the software packages BAS Reader (version 3.14) and AIDA (version 3.25; Raytest). Protein concentration was determined according to Bradford (1976).

### 2.8 Statistical methods

The data were submitted to Student's *t* test for statistically significant difference (*p < .05; **p < .01). Results are shown as mean ± *SE* with at least three biological replicates.

## 3 RESULTS

### 3.1 WHIRLY2 affects mitochondria morphology in cultured cells

To evaluate the role of WHIRLY2 in Arabidopsis, the *why 2‐1* mutant line, previously described as a true knockout mutant (Janicka et al., 2012), harboring the T‐DNA insertion in the last intron of *WHIRLY2*, was used.

**FIGURE 1:**
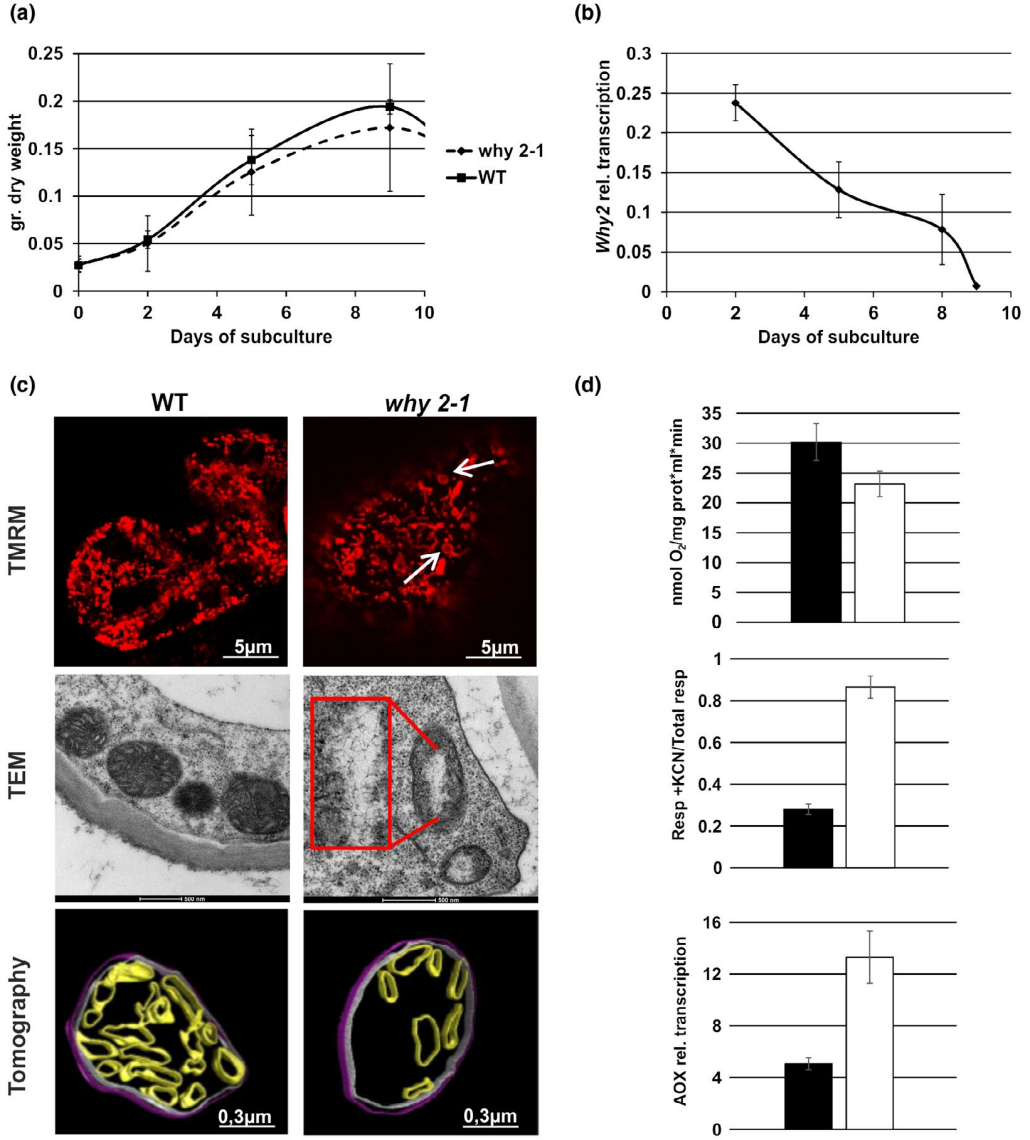
WT and *why 2‐1* Arabidopsis suspension cell cultures. (a) Growth curve calculated as dry weight (gr.) at different days after subcultures. (b) *Why 2* expression profile in WT suspension cell cultures at different days after subcultures. (c) Confocal, transmission electron microscope images and tomographic 3D reconstruction model of mitochondria from suspension cell cultures at 5 days after subcultures. Inset magnification 3X. Arrows in the CLSM image indicate mitochondria with peculiar morphology. Different colors were used for the rendering of the different suborganellar structures in the tomographic 3D reconstruction: magenta for inner membranes (IM), blue for outer membranes (OM), and green for cristae. (d) Mitochondria functionality in WT and *why 2‐1* suspension cell cultures defined as: Oxygen consumption (upper plot), alternative oxidase capacity (middle plot) of cells treated with 1 mM KCN, relative expression profile of the alternative oxidase (AOX; lower panel). Values represent the mean ± standard deviation of three independent experiments performed in triplicate. The asterisks indicate values that are significantly different from WT cells using the Student's *t* test method (*p < .05)

Cell suspension cultures were generated from both WT and *why 2‐1* plants. Such cultures represent an ideal system for detailed analysis at the cellular level, being homogeneous, fast growing, easy to handle, and readily accessible to treatments. WT and *why 2‐1* cell cultures were characterized with regard to their dry weight (Figure 1a) showing no considerable differences between the two lines. The expression level of *Why 2* was determined at different stages of culture growth and it appeared to be high in the early subculture phase and decreased during cell culture progression (Figure 1b).

With regard to the mitochondrial localization of WHIRLY2, the impact of *Why 2* disruption on mitochondria morphology, ultrastructure, dynamics, and functionality was investigated. CLSM imaging of cultured cells stained with TMRM allowed to evaluate mitochondria morphology in vivo (Figure 1c). While, in the WT line, mitochondria appear to be spherical or punctiform, mutant lines had an impaired mitochondrial morphology whereby mitochondria appeared as more elongated and sometimes showed a peculiar loop shape (Figure 1c, arrows) that in animal system has been demonstrated to be typically associated with cellular oxidative stress conditions (Jaipargas, Barton, Mathur, & Mathur, 2015). TEM images revealed that disruptive rearrangements have taken place in the mitochondria of *why 2‐1* cell lines. While in WT cell lines mitochondria were round or oval, contained several cristae and an electron‐dense matrix, mitochondria in the *why 2‐1* lines, instead, appeared to be swollen, with a reduced number of cristae and a low electron density matrix that might indicate a low functionality (Logan, 2006;Vigani et al., 2015). Interestingly, a large translucent area (Figure 1c inset) in the center of the organelles is present, where fibrillar structures resembling unpacked DNA are evident.

In order to investigate the ultrastructure of mitochondria in the cells of the *why 2‐1* mutant line, representative specimens were selected for tomographic reconstructions as described by Vigani et al. (2015). At the ultrastructural level, mitochondria from the WT cell culture displayed homogenous matrices as well as regular internal cristae (Figure 1c). In contrast, in the mutants, a heterogeneous morphology of mitochondria was observed. While few of them appeared to be only slightly altered, the majority of mitochondria had a lower matrix density and a reduced number of cristae when compared to WT plants. Such ultrastructural alterations were observed in all the examined samples from three independent experiments. The numbers of cristae as well as the sizes of the relative intracristae surface areas were determined on mitochondria randomly selected for each sample (Talbe S2). In the *why 2‐1* mutant line, the number of cristae per mitochondrion in cultured cells was reduced by about 20%, while the relative intracristae surface area (intracristae surface area per mitochondrion) was even reduced by about 40% (*p* < .05; Table S2). These data taken together strongly support a key role of WHIRLY2 in nucleoid structure maintenance and in shaping the morphology of the mitochondria.

### 3.2 WHIRLY2 affects the functionality of mitochondria in cultured cells

The observed changes in the morphology of mitochondria might lead to changes in the functionality of these organelles. Therefore, respiratory efficiencies of WT and mutant cell lines were compared. The total respiration of *why 2‐1* cells is reduced by 30% compared to WT (Figure 1d, upper plot). Seventy percentage of the residual oxygen consumption present in the *why 2‐1* mutant has to be assigned to the alternative pathway, defined as AOX capacity (Figure 1d, middle plot). AOX capacity is defined as the part of the O_2_ consumption that is insensitive to the cytochrome (*cyt*) pathway inhibitor KCN, and sensitive to the AOX inhibitor (salicylic‐hydroxamic acid, SHAM). This measure of capacity is typically related to the abundance of AOX. Specific AOX gene family members are strongly induced at the transcript and protein level by an insufficient *cyt* pathway capacity downstream of the ubiquinone pool (Vanlerberghe, Martyn, & Dahal, 2016;Yu, 2019). Accordingly, in the *why 2‐1* mutant cells, the expression level of the AOX gene is higher than in WT cells (Figure 1d, lower plot), thus supporting the data on an increased AOX capacity observed in the mutant line.

### 3.3 WHIRLY2 localizes to mitochondria in different plant organs

**FIGURE 2:**
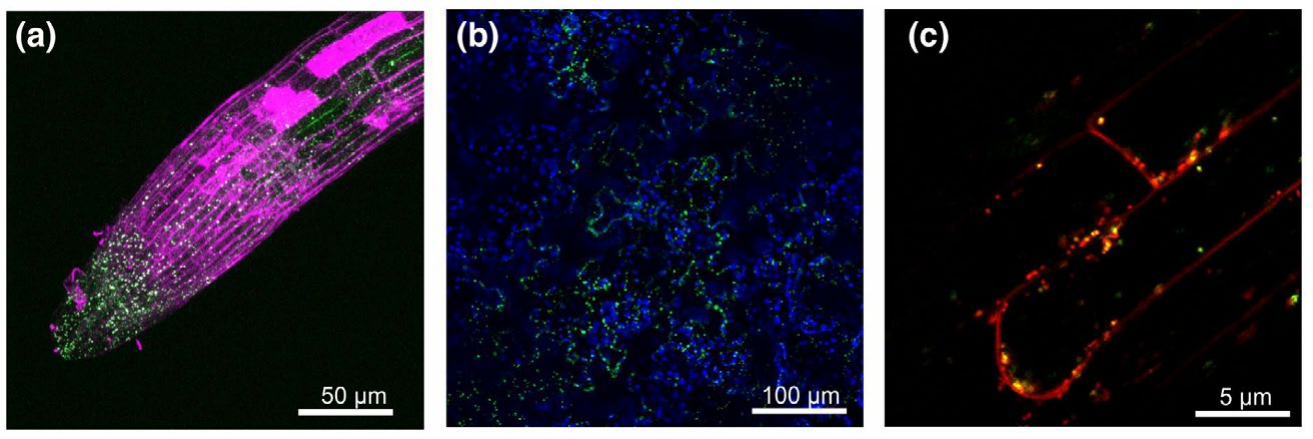
Mitochondrial localization pattern of *35S‐WHIRLY2:GFP* in roots (a) stained with propidium iodide (magenta), leaf epidermal cells (b; blue color is due to chlorophyll), and hypocotyl (c) stained with TMRM (red)

In order to verify the mitochondrial localization of WHIRLY2 *in planta*, stable transformed Arabidopsis plants harboring the WHIRLY2 coding sequence fused to the GFP gene under control of the 35S CaMV promoter were generated. In stable transformed plants, GFP fluorescence was detected in different organs, in roots (Figure 2a), in leaf (Figure 2b), and in hypocotyl (Figure 2c), as small and highly dynamic punctate structures (Movie S1). In the root, WHIRLY2 appears to be more concentrated at the level of the root tip most likely because of the small size of cells, as compared to elongated cells whose volume is mostly occupied by the vacuole. In Figure 2c, it is shown that WHIRLY2:GFP clearly colocalize with the mitochondria‐specific dye TMRM confirming the mitochondrial localization previously determined in transiently transformed protoplasts (Krause et al., 2005). Within the mitochondria, WHIRLY2:GFP fluorescence was not equally distributed but appeared in punctiform spots. It has also been observed that discrete WHIRLY2:GFP spots move between mitochondria during the fusion–fission processes (Movie S1). All these data are consistent with a putative nucleoid association of WHIRLY2, supporting a role in mtDNA packaging/maintenance. Another interesting issue is that WHIRLY2 is not equally present in all the mitochondria stained by TMRM and not even uniformly distributed in the root tip where WHIRLY2 appears to be more concentrated at the level of the tip (Figure 2a).

### 3.4 *why 2‐1* mutant plants have an altered mitochondrial structure

**FIGURE 3:**
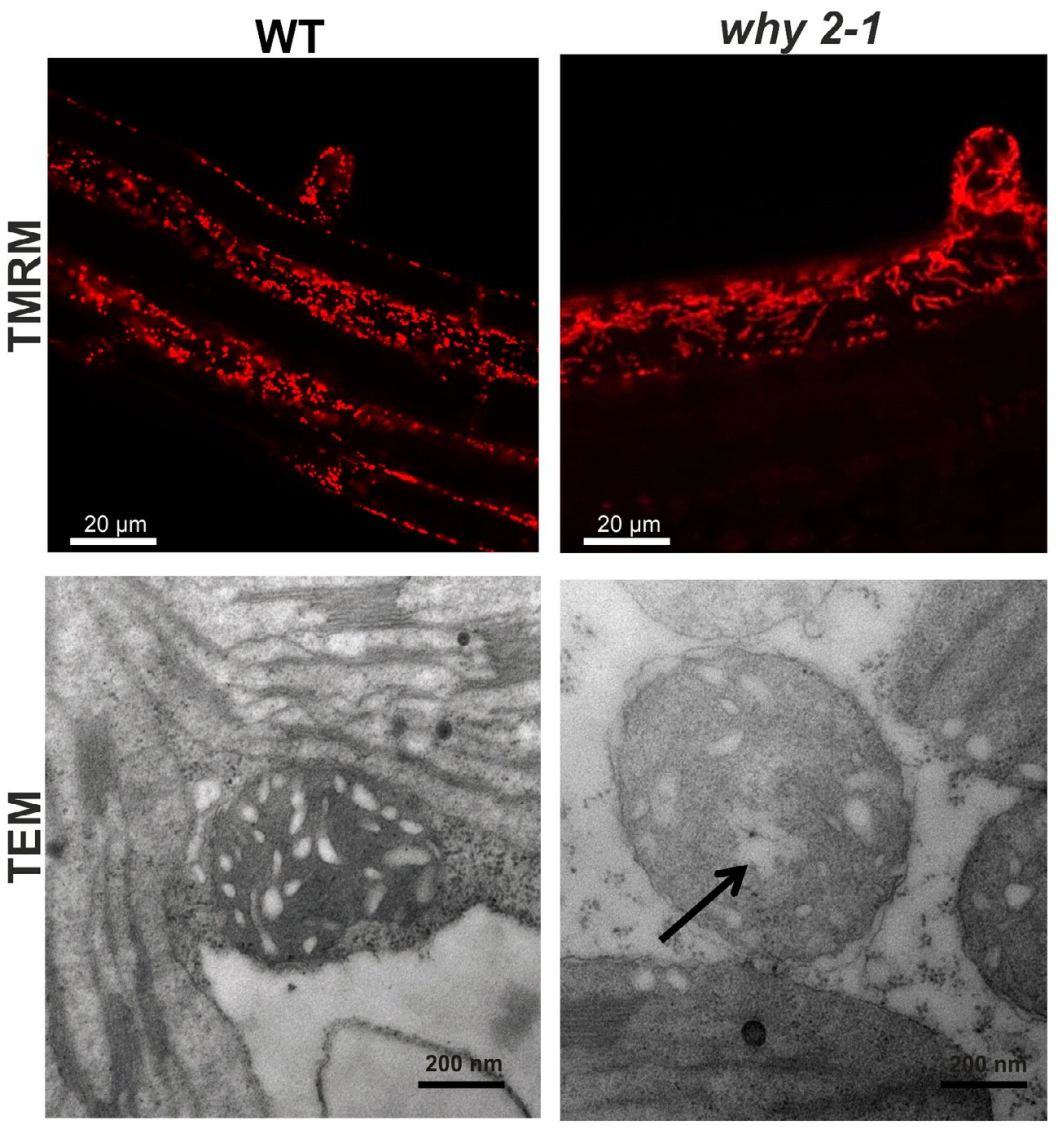
Mitochondria morphology in plant tissues. Upper panel: confocal images of roots of WT and *why 2‐1* plants stained with TMRM. Lower panel: transmission electron microscope images of mitochondria from leaf section from 3‐week‐old WT and mutant plants. Arrow indicates translucent area within mitochondria matrix

In order to compare the mitochondria morphology in WT and mutant plants in vivo, Arabidopsis roots were stained with TMRM and analyzed by confocal microscopy. In mutant lines, mitochondria appear as elongated organelles (Figure 3 upper panel) characterized by reduced dynamics when compared with those present in the roots of WT plants (Movie S2 and S3). On leaf sections from 3‐week‐old plants of Col‐0 and *why 2‐1* mutant TEM analyses were performed as indicated in Figure 3 (lower panel). In the mutant line, mitochondria resemble those identified in cultured cells confirming the swollen morphology, the reduced number of cristae, and the presence of a translucent area (arrow) within the mitochondrial matrix. Approximately 30% of the mitochondria in leaves of the mutant line exhibited such altered morphology compared with the WT (Figure 3 lower panel). Furthermore, as calculated from TEM images of leaf sections, the relative intracristae surface area decreased by about 40% (*p* < .05) in the *why 2‐1* mutant line when compared to the WT (Table S2).

### 3.5 Seed germination is compromised in *why 2‐1* mutants

**FIGURE 4:**
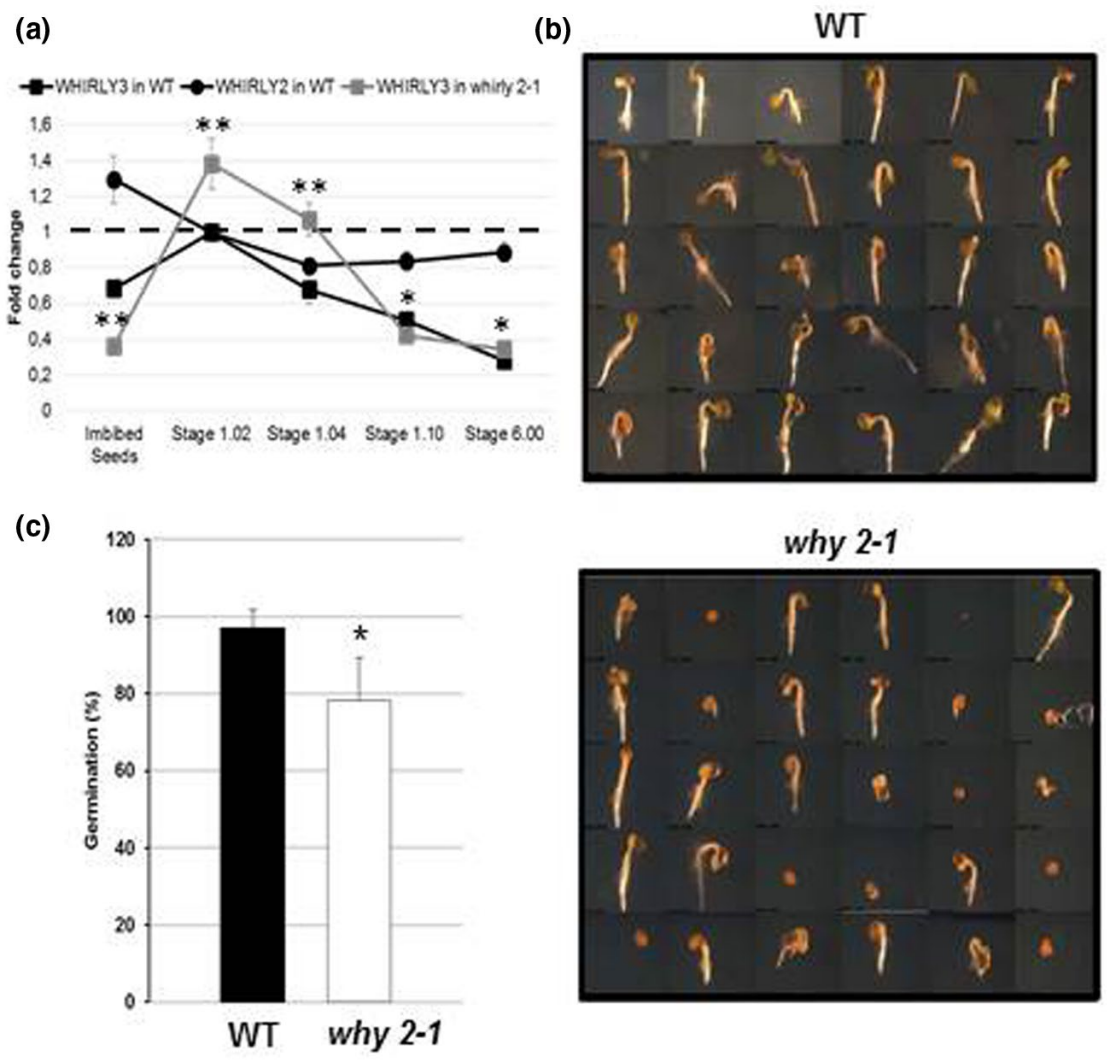
Germination assay of WT and *why 2‐1* mutant. (a) Expression profile of *Why 2* and *Why 3* genes in WT and *why 2‐1* plants and seeds. The expression was analyzed in 24 hr imbibed seeds and in plants at 1.02, 1.04, 1.10, and 6.00 stages of growth. Data were analyzed using the ΔΔCT method. Values represent the mean ± confidence interval (*p* < .05) of three independent experiments performed in triplicate. (b) Representative stereomicroscope images of WT and *why 2‐1* seedlings 4 days after sowing (DAS); (c) Percentage of germinated seed at 4 DAS

In Figure 4a, the expression profile of *WHIRLY2* in different developmental phases, from imbibed seeds to flowering stage, is reported. The results show a relatively high level of the expression in 24 hr imbibed seeds, as expected for proteins involved in organelle DNA repair/replication in rapidly growing tissues (Diray‐Arce, Liu, Cupp, Hunt, & Nielsen, 2013), with a subsequent drop in expression level.

In order to investigate whether the presence of aberrant mitochondria in the *why 2‐1* mutant could have consequences for development of the plant, WT and *why 2‐1* plants were compared at different stages of development, starting from seed germination. Sterilized seeds were sown in solid one‐half MS medium, stratified for 48 hr, and, then, placed in a growth chamber. Seeds were microscopically analyzed to evaluate the rupture of the *testa* indicating the beginning of germination process (Figure 4b). A clear difference between the two genotypes was detected: *why 2‐1* seeds showed a significant reduction (20%) in the percentage of germination compared to the WT (Figure 4c). These data highlight the important role of WHIRLY2 in a phase of plant life characterized by active mtDNA synthesis, such as seed germination (Paszkiewicz, Gualberto, Benamar, Macherel, & Logan, 2017).

### 3.6 WHIRLY3 mRNA level and protein abundance are enhanced in shoots of the *why 2‐1* mutant

The "mild" development‐dependent phenotype of the *why 2‐1* mutant suggests that in adult mutant plants, where no phenotype is shown, the lack of the WHIRLY2 protein could be compensated, at least in part. A possible candidate for such functional compensation could be another WHIRLY protein. In the absence of WHIRLY2 an increase in the mRNA level of WHIRLY3 was observed at earlier stages of shoot development (Stage 1.02; Figure 4). The low expression level of *WHIRLY3* is, instead, not altered in imbibed seeds. These data suggest a tight coordination between the expression of *Why 2* and *Why 3*.

### 3.7 The WHIRLY3 protein is imported into chloroplasts as well as into mitochondria

Functional compensation of absence of WHIRLY2 is also a matter of the correct subcellular localization, i.e., WHIRLY3 must be present in the same subcellular compartments to be capable of compensating the lack of WHIRLY2. However, upon transient transformation of suitable GFP‐fusion polypeptides in tobacco protoplasts, the WHIRLY2 protein was shown to accumulate in mitochondria, while WHIRLY3 could be detected solely in chloroplasts (Krause et al., 2005), which makes a functional compensation rather unlikely. On the other hand, *in organello* protein transport experiments performed with WHIRLY2 clearly demonstrated dual targeting properties of the protein, i.e., the authentic precursor is imported into both, chloroplasts and mitochondria, when incubated with isolated intact organelles (Krause et al., 2005). Such complementing *in organello* transport experiments have not been carried out though with WHIRLY3.

**FIGURE 5:**
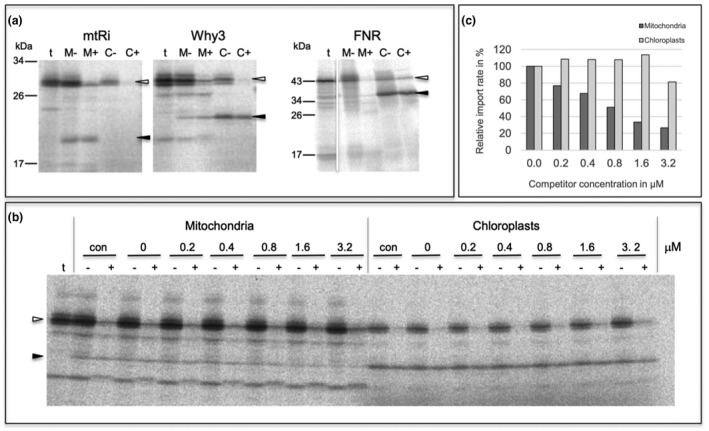
*In organello* protein transport experiments with isolated pea organelles. (a) Radiolabeled precursor polypeptides of mitochondrial Rieske Fe/S protein (*mtRi*), WHIRLY3 (*Why3*), and chloroplast Ferredoxin‐NADP‐Oxidoreductase (*FNR*) were obtained by coupled in vitro transcription/translation of the corresponding cDNA clones and incubated for 20 min at 25°C with either intact mitochondria (*lanes M*) or chloroplasts (*lanes C*) from pea. After the import reaction, the organelles were recovered by centrifugation and treated with thermolysin (*lanes M+*, *C+*) or mock treated (*lanes M‐, C‐*). Stoichiometric amounts of each fraction corresponding to 50 µg protein (mitochondria) or 12.5 µg chlorophyll (chloroplasts) were separated on a 10%–17.5% SDS polyacrylamide gradient gel and visualized by phosphorimaging. In *lanes t*, 1 µl aliquots of the in vitro translation assays (corresponding to 10% of the protein added to each import reaction) were loaded. The position of precursor and mature polypeptides are indicated by *open arrowheads* and *filled arrowheads*, respectively. The size of molecular marker proteins is given in kDa. (b) Effect of competitor protein on organelle import of WHIRLY3. *In organello* protein transport experiments with the WHIRLY3 precursor protein were performed in the absence (*con*) or presence of increasing amounts of the precursor of mitochondrial Rieske Fe/S‐protein which was obtained by heterologous overexpression in *Escherichia coli*. The concentration of competitor protein present in each assay (given in µM) is indicated above the lanes. (c) Bar chart showing the relative amounts of WHIRLY3 accumulating in the organelles of the competition experiment shown in (b). The bands corresponding to mature WHIRLY3 polypeptide in lanes + were quantified and depicted in terms of percentage of mature WHIRLY3 in the control reaction in the absence of competitor (lanes 0)

Therefore, *in organello* protein transport experiments using intact mitochondria and chloroplasts that were isolated from a single pulping of pea leaves (Rödiger et al., 2010) were performed here with the authentic precursor of WHIRLY3. In addition to WHIRLY3, two control proteins with known organelle localization, namely, chloroplast FNR (Ferredoxin‐NADP‐Oxidoreductase) and the mitochondrial Rieske‐Fe/S protein (mtRi), were analyzed in parallel. For this purpose, freshly isolated intact organelles were incubated with the respective radiolabeled precursor proteins, which were obtained by in vitro transcription of the corresponding cDNA clones and subsequent in vitro translation with reticulocyte lysates in the presence of [35S]‐methionine. The two control proteins showed the expected organelle specificity: mtRi is imported into mitochondria but not into chloroplasts, while FNR shows the reciprocal result, i.e., it is transported into chloroplasts but not into mitochondria (Figure 5). Within the organelles, the two precursor proteins are cleaved by organellar processing peptidases to yield the respective mature proteins. These processing products are resistant to protease added externally to the assays after import, which confirms that they have been internalized into the organelles (Figure 5). In contrast, precursor proteins that are not imported but remain attached to the organellar envelopes in the course of the experiment are largely, although not always completely, degraded under these conditions.

Remarkably, WHIRLY3 is not only imported into chloroplasts but also accumulates in a protease‐protected manner also in mitochondria (Figure 5). Within both organelles the protein is processed after membrane transport to a product of approximately 24 kDa, which corresponds well to the size of the mature protein (Isemer et al., 2012). In contrast, the control protein for chloroplast import, FNR, does not show any hint of mitochondrial import in these assays which rules out that the mitochondrial import of WHIRLY3 observed is due to contaminating chloroplasts in the mitochondrial preparation.

This was further confirmed by competition experiments that were performed as a complementary approach to evaluate the dual targeting properties of WHIRLY3. In these experiments, the mitochondrial import machinery was gradually saturated by excess amounts of the mitochondrial control protein, mtRi, which was obtained by overexpression in *Escherichia coli*. With increasing concentration of mtRi precursor in the assay, import of WHIRLY3 into mitochondria decreased gradually (Figure 5). Control experiments showed that the import of WHIRLY3 into chloroplasts remained unaffected in the presence of the competitor protein, which emphasizes the specificity of the competition reaction.

## 4 DISCUSSION

WHIRLY2 is a member of the small plant‐specific family of WHIRLY proteins binding to single stranded DNA and proposed to be key components of the organelle repair machinery (Cappadocia et al., 2010;Marechal et al., 2009). It has been reported that WHIRLY2 and other plant‐specific single‐stranded DNA binding proteins hinder microhomology‐mediated end‐joining (MMEj; García‐Medel et al., 2019). In the present study, it has been demonstrated that WHIRLY2 deficiency compromises mitochondria ultrastructure, morphology, and functionality. In *why 2‐1* mutants, mitochondria contain a low number of cristae and show a reduced functionality if compared to the WT, as evidenced by the reduced respiration activity and the low electron density of the organelles analyzed by TEM. TEM analysis showed that *why 2‐1* mutant mitochondria house a peculiar translucent area likely containing filamentous mtDNA. This phenomenon resembles what has been already observed in WHIRLY1 knockdown plants of barley where translucent areas in chloroplasts from WHIRLY1‐deficient plants coincide with reduced packaging of nucleoids (Krupinska et al., 2014). The data presented in this study confirm the nucleoid localization of WHIRLY2 but also support a structural role in mitochondria nucleoid organization. The alteration of the shape of mitochondria toward swollen organelles, observed in *why 2‐1* plants, is likely due to an impairment in the recruitment of the fission proteins involved in mitochondria division (Yan, Duanmu, Zeng, Liu, & Song, 2019) while the elongation could be attributed to a perturbation of the fusion/fission balance. These data strongly suggest that a tight association exist between nucleoid organization and mitochondria morphology and dynamics. Besides, in mammals, it has been demonstrated that the integrity of mtDNA/nucleoids plays an important role in the remodeling of cristae structures (Ban‐Ishihara, Ishihara, Sasaki, Mihara, & Ishihara, 2013). Moreover, it has been suggested that, in plants, mitochondrial dynamics, that reshape organelle morphology through the ongoing fusion and fission events, is an important feature for maintaining mitochondrial plasticity but also to provide a means for promoting recombination of DNA fragments (Arimura, 2018). MMF is likely to facilitate nucleoid transmission, mitochondrial DNA (mtDNA) recombination, and the homogenization of mitochondrial components, thus providing a type of quality control for mitochondrial populations (Rose & McCurdy, 2017).

It has been demonstrated that in *why 2‐1* plants the microhomology‐mediated DNA rearrangements (MHMRs) occur at higher levels than in the WT. It is likely that the accumulation of DNA rearrangements could be responsible for nucleoid disorder and consequently for the elongated mitochondria. The observed reduced mitochondrial dynamics would in turn favors mitochondria fusion and elongation. The alterations in mitochondrial morphology and functionality observed in the *why 2‐1* mutant are expected to have a severe impact on development and growth of the plants. For this reason, an impairment of growth is expected. On the other hand, no obvious phenotype is evident in the *why 2‐1* mutant plants during vegetative growth (Maréchal et al., 2008). Indeed a significant decrease in seed germination percentage was observed in *why 2‐1* compared to WT background. A possible explanation might be that the impact of WHIRLY2 absence is particularly pronounced when organelle DNA synthesis is active, such as in highly dividing cultured cells and during germination (Arimura, 2018;Cheng et al., 2017).

During germination a metabolic reorganization occurs: the higher expression of WHIRLY2 was observed in the 24h imbibed seeds when reactivation of cellular and mitochondrial metabolism occurs (Paszkiewicz et al., 2017). It must be taken into consideration that mtDNA replication probably takes place by recombination‐dependent replication occurring as a result of double‐stranded homologous recombination breakage or of double‐ or single‐stranded break repair mechanisms that likely involve WHIRLY2 (Cheng et al., 2017).

The lack of an evident mutant phenotype of the mature plant upon abrogation of *Why 2* expression suggests the presence of functional homologues or the activation of compensating mechanisms in mitochondria. Possible candidates for compensation of WHIRLY2 deficiency in the *why 2‐1* mutant are WHIRLY1 and WHIRLY3. WHIRLY1 was shown to be exclusively imported into chloroplasts both in *in organello* protein transport experiments and after transient transformation of protoplasts (Krause et al., 2005) which precludes a functional role in mitochondria. WHIRLY3 was likewise found solely in chloroplasts after transient expression of a reporter construct (Krause et al., 2005). However, this result was not independently confirmed with a second method which was shown to be essential to prevent potential misinterpretation (Sharma, Bennewitz, & Klösgen, 2018). Indeed, as proven here by *in organello* import as well as competition experiments, WHIRLY3 is dually targeted into both, mitochondria and chloroplasts (Figure 5), which in principle enables the compensation of WHIRLY2 deficiency by WHIRLY3. In line with that, *Why 3* expression is actually higher in the *why 2‐1* mutant than in the WT at early stages of development.

In a similar scenario, WHIRLY3 might even compensate for WHIRLY1 deficiency considering the lack of an apparent mutant phenotype of the *why1* knockout mutant (Yoo, Kwon, Lee, & Chung, 2007). In contrast, in plants lacking WHIRLY3 such as maize and barley, WHIRLY1 deficiency leads to a disturbance of chloroplast development (Krupinska et al., 2019;Prikryl, Watkins, Friso, van Wijk, & Barkan, 2008).

Although further efforts are required to elaborate the functional interaction of WHIRLY proteins, these data suggest already that the coordination of the three WHIRLY proteins in *A. thaliana* is required to guarantee the integrity of the organellar genomes in the early phases of seed germination.

## 5 CONCLUSIONS

In this study, we identify a role of WHIRLY2 in maintaining mitochondria morphology and functionality. In particular, we found that the absence of WHIRLY2 is associated with a disorganization of the nucleoids and the fine ultrastructure of the mitochondria both in cultured cells and in leaf. We demonstrated that the strong mitochondria alterations observed in the *why 2‐1* mutant significantly compromise the seed germination process. Experiments by means of an *in organelle* import assay demonstrate that along with WHIRLY2 also WHIRLY 3 can enter the mitochondria, strongly suggesting a compensatory activity of WHIRLY3 in *why 2‐1* mutant plants. These results cast new light on the role of the mitochondrial protein WHIRLY2 in plant cells paving the way for further studies on the link between mitochondria structure/morphology and functionality both at the cellular and at the plant level.

## AUTHOR CONTRIBUTIONS

MZ and KK conceived the project, MZ designed the experiments and analyzed the data; SG and YLN performed most of the experiments on Arabidopsis plants and cell cultures; NLR and MM performed the TEM experiments; BB and RBK designed and performed the experiments of *in organello* assay; GV with the technical assistance of FC performed the tomography experiments; MZ and KK wrote the article with contributions of all the authors; FLS supervised and completed the writing; MZ agrees to serve as the author responsible for contact and ensures communication.

## Supporting information

Table S1‐S2

Movie S1

Movie S2

Movie S3
