# Supplementary material for: WHIRLY2 plays a key role in mitochondria morphology, dynamics, and functionality in Arabidopsis thaliana
Source: Plant Direct. 2020 May 30;4(5):e00229. doi: 10.1002/pld3.229 (PMC7261051; doi:10.1002/pld3.229)
Supplement: Supplementary file 1 — Table S1‐S2 [file PLD3-4-e00229-s001.docx]

**WHIRLY 2 plays a key role in mitochondria morphology, dynamics and functionality in *Arabidopsis thaliana***

Serena Golin^a^, Yuri Luca Negroni^a^, Bationa Bennewitz^b^, Ralf Bernd Klösgen^b^, Maria Mulisch^c^, Nicoletta La Rocca^a^, Francesca Cantele^d^, Gianpiero Vigani^e^, Fiorella Lo Schiavo^a^, Karin Krupinska^c^, Michela Zottini^a^

**Supplemental material**

| **Supplementary Table 1.** Primer List | |
| --- | --- |
| **Primer Name** | **Primer Sequence** |
| act2_fwd | AAGCTCTCCTTTGTTGCTGTT |
| act2_rvs | GACTTCTGGGCATCTGAATCT |
| why2‐fwd | GCATCCTCAAAACCAATGAC |
| why2‐rvs | CATGATGTGTGGAAGAGCAA |
| why3‐fwd | ACGATAGAACCACGAGCACCAG |
| why3‐rvs | TGTCAGCTTGAACGCACCAGATTC |
| AOX1a_RT‐fwd | TGGTTGTTCGTGCTGACG |
| AOX1a_RT‐rvs | CACGACCTTGGTAGTGAATATCAG |

| **Supplementary Table 2.** Determination of the cristae number per mitochondrion and the cristae area (intracristae area) per mitochondria area (expressed as % ratio). Mean values ± SD are from analysis of around 20 mitochondria randomly selected from inclusions obtained from 3 independent biological samples. Student t-test was adopted to analyse statistical significance (*p*<0,05) with respect to wild type (WT). | | | | |
| --- | --- | --- | --- | --- |
|  |  | **WT** | ***why 2-1*** | ***p*** |
| Plants | Number of cristae /mitochondrion | 14,43 ± 2.41 | 12.83 ± 3.83 | *0,401* |
|  | Cristae area/mitochondria area (%) | 24,26 ± 7,34 | 13.77 ± 5.77 | ***0,012*** |
| Cells | Number of cristae /mitochondrion | 11,00 ± 2,87 | 8.86 ± 2.96 | *0,090* |
|  | Cristae area/mitochondria area (%) | 19,75 ± 6,30 | 11.58 ± 4,23 | ***0,0009*** |
