# Supplementary figures and images for: WHIRLY2 plays a key role in mitochondria morphology, dynamics, and functionality in Arabidopsis thaliana
Source: Plant Direct. 2020 May 30;4(5):e00229. doi: 10.1002/pld3.229 (PMC7261051; doi:10.1002/pld3.229)

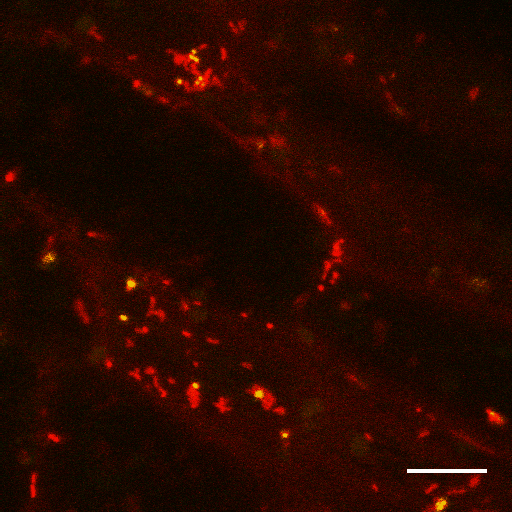

Supplement: Supplementary file 2 — Movie S1 [file PLD3-4-e00229-s002.gif]

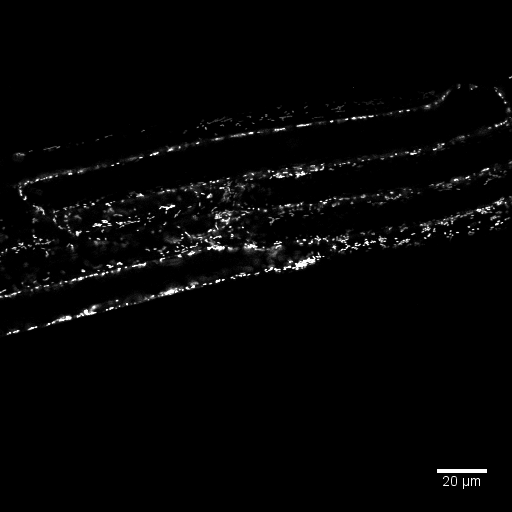

Supplement: Supplementary file 3 — Movie S2 [file PLD3-4-e00229-s003.gif]

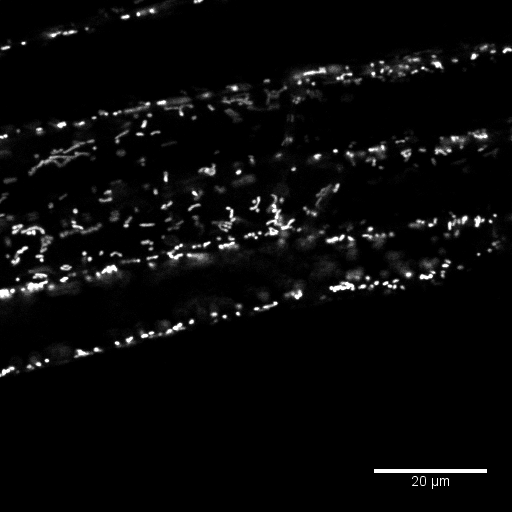

Supplement: Supplementary file 4 — Movie S3 [file PLD3-4-e00229-s004.gif]
